# Co-creating mental health promotion and prevention interventions with groups in vulnerable situations in Europe: a mixed-methods study protocol for co-creation implementation and participatory evaluation

**DOI:** 10.1186/s12961-025-01349-1

**Published:** 2025-07-01

**Authors:** Raquel Teixeira, Cláudia de Freitas, Liuska Sanna, Eleonora Prina, Giulia Turrini, Amber S. Brizar, Cătălin Gherdan, Evaldas Kazlauskas, Austėja Dumarkaitė, Henrik Wasmus, Leonie Fleck, Ulrich Reininghaus, Melanie Mack, Chiara Scarampi, Maritta Välimäki, Maija Satamo, Wietse A. Tol

**Affiliations:** 1 EPIUnit ITR, Institute of Public Health of the University of Porto, University of Porto, Porto, Portugal; 2 Mental Health Europe, Brussels, Belgium; 3 WHO Collaborating Centre for Research and Training in Mental Health and Service Evaluation, Department of Neuroscience, Biomedicine and Movement Sciences, Section of Psychiatry, University of Verona, Verona, Italy; 4 Department of Experimental and Applied Psychology, Institute for Brain and Behavior Amsterdam, Vrije Universiteit Amsterdam, Amsterdam, The Netherlands; 5 Department of Clinical, Neuro- and Developmental Psychology, Amsterdam Public Health Research Institute, Vrije Universiteit Amsterdam, Amsterdam, The Netherlands; 6 Center for Psychotraumatology, Institute of Psychology, Vilnius University, Vilnius, Lithuania; 7 Department of Public Mental Health, Central Institute of Mental Health (CIMH), Medical Faculty Mannheim, Heidelberg University, Mannheim, Germany; 8 Health Service and Population Research Department, Institute of Psychiatry, Psychology & Neuroscience, King’s College London, London, United Kingdom; 9 Center for the Interdisciplinary Study of Gerontology and Vulnerabilities (CIGEV), University of Geneva, Geneva, Switzerland; 10 Swiss Centre of Expertise in Life Course Research, LIVES Centre, Lausanne, Switzerland; 11 School of Public Health and Helsinki University Hospital, University of Helsinki, Helsinki, Finland; 12 Department of Nursing Science, Faculty of Medicine, University of Turku, Turku, Finland; 13 Department of Public Health, University of Copenhagen, Copenhagen, Denmark; 14 Athena Research Institute, Vrije Universiteit Amsterdam, Amsterdam, The Netherlands; 15 German Center for Mental Health (DZPG), Partner Site Mannheim-Heidelberg-Ulm, Mannheim, Germany; 16 Swiss Centre of Expertise in Life Course Research, LIVES Centre, Geneva, Switzerland; 17 Faculty of Psychology and Education Sciences, University of Porto, Porto, Portugal; 18 Center for Psychology, University of Porto, Porto, Portugal

**Keywords:** Mental health, Primary prevention, Public health, Co-creation, Stakeholder participation, Evaluation

## Abstract

**Background:**

Co-creation has emerged as a crucial strategy for addressing complex public health challenges, including promotion and prevention of mental health concerns. While the evidence base for effective interventions continues to grow, significant gaps remain in their implementation and integration into real-world settings. Co-creation offers a valuable tool for strengthening mental health promotion strategies, ensuring that interventions are evidence-based, contextually relevant, culturally sensitive, sustainable and acceptable to those directly affected. However, there is a paucity of studies examining the evaluation of co-creation research, particularly regarding how participatory methods foster adaptation and influence outcomes and long-term sustainability. This protocol outlines a study designed to implement, evaluate and strengthen co-creation methodologies through a participatory and formative evaluation approach.

**Methods:**

This study adopts a mixed-methods design within the ADVANCE project, a multi-country initiative focused on co-creating mental health promotion and prevention interventions with groups in vulnerable situations across seven European countries. End-users, healthcare professionals, and decision-makers will be engaged throughout the project in both intervention design and evaluation. Co-creation activities initiated with intervention scenario building and prioritization, drawing on desk reviews and online Delphi surveys co-developed with locally-set Society Advisory Groups (SAGs). The selection of intervention scenarios for implementation was performed using scenario-based workshops involving stakeholders in six partner countries. A second goal is to evaluate the co-creation process, which was co-designed in consultation with country teams and SAGs. A longitudinal qualitative study based on semistructured interviews with co-creators across two time points will be conducted, following the co-development of the interview guide through an online World Café.

**Discussion:**

This study introduces an innovative approach by embedding participatory and formative evaluation into the co-creation process, enabling ongoing adaptation of co-creation activities. Through continuous stakeholder engagement, the project seeks to address barriers deriving from power imbalances, conflicting priorities, and resource limitations. Qualitative and participatory methods will be combined to elicit stakeholders’ views, identify drawbacks and promote adjustments to ensure meaningful collaboration and reduce participation fatigue. Expected outcomes include actionable recommendations to inform policy, reduce stigma and foster the co-creation of more inclusive, effective, sustainable and scalable mental health promotion and prevention strategies across Europe.

## Introduction

Co-creation in research has been advocated as an important strategy for tackling so-called wicked problems, i.e. societal challenges influenced by complex social and political factors [1–3]. Increased mental health concerns in Europe have been described as a wicked problem [2]. Mental health issues affect more than one in six people in the European region, constituting 26% of the overall burden of disease [4, 5]. Anxiety, depression and substance abuse are on the rise due to a multitude of factors, including demographic shifts, economic downturns, climate change, digitalisation, conflict and migration [6]. Consequently, large groups of people are currently identified as being at higher risk, and mental health care systems across Europe are experiencing great pressure to meet demands while facing a provider shortage [7, 8]. This has prompted decision-makers, healthcare providers and researchers to prioritize the development of effective strategies aimed at promoting mental well-being and preventing mental health issues in co-creation with diverse communities in Europe [9, 10].

Various mental health promotion and prevention strategies have been shown to effectively strengthen positive aspects of mental health and reduce the incidence of mental health concerns [11–13]. However, knowledge of intervention effectiveness is not sufficient to guarantee seamless integration into healthcare practice, organizational structures and policy contexts. Significant gaps between scientific evidence on effective interventions and their actual implementation in real-world settings have been documented [14–16]. Dissemination and implementation (D&I) science is an evolving discipline that seeks to bridge these gaps with a range of methods and strategies to promote the uptake of evidence-based interventions into routine practice [15, 17]. Best practices in D&I science underscore the significance of adopting transdisciplinary and co-creation approaches, engaging decision-makers (e.g. politicians and regulators) and researchers from diverse fields, collaborating with local community partners actively involved in on-the-ground initiatives, and incorporating the insights of individuals directly affected by the interventions (i.e. patients, clients and end-users) [16, 18, 19].

Engaging in co-creation in the mental health field can provide an inclusive, collaborative and iterative perspective on implementing and assessing evidence-based interventions. By ensuring that all stakeholders have an equal voice in the process, co-creation aims to enhance the relevance, ownership, uptake and sustainability of health interventions while promoting health equity and fostering transformative changes [19–21]. Furthermore, co-creation may contribute to reducing stigma surrounding mental health by challenging stereotypes and fostering empathy and solidarity within communities [22, 23]. In light of historical breaches of the principle of respect for individuals in mental health research, their involvement has been argued as a reparative act, an attempt to restore justice and balance out the uneven distribution of power [22, 24]. Importantly, involving people with lived experience as co-researchers in mental health studies should extend beyond the recognition and validation of experiential knowledge to direct engagement in research evaluation to improve its overall outcomes. However, despite its promising results, only a handful of studies have engaged in participatory co-creation research evaluation [25–29]. This can be partly attributed to co-creation, which involves multiple stakeholders with distinct perspectives and goals, presenting significant methodological challenges for designing evaluations that effectively capture both the process and its outcomes across a diverse array of co-creators [29]. Evaluating co-creation can also surface tensions related to power sharing, ownership and the critiquing of existing structures, potentially generating discomfort and discouraging deep engagement in evaluation [30]. Finally, there are is no widely adopted framework for evaluating co-creation in health. However, recent efforts to systematize and adapt evaluation frameworks from public health interventions to co-creation research represent a promising step towards reducing resource constraints and other persistent barriers to effective evaluation [31].

The benefits described above position co-creation as an invaluable tool for strengthening mental health promotion and prevention strategies. Although literature highlights commendable efforts in the mental health domain [32, 33], persisting challenges in this field call for innovative approaches that can promote early, equitable and consistent involvement of service users together with other stakeholders to advance the field of mental health promotion and prevention across the lifespan [34]. These approaches must also prioritise formative evaluation of co-creation, i.e. evaluation that starts early on in the process and carries through iteratively to identify scope for improvement and facilitate co-creation adaptation towards maximal acceptability and responsiveness [27, 35, 36]. Formative co-creation evaluation is especially critical when partnering with groups who have been traditionally disenfranchised from participatory health fora and who have lacked the opportunity to influence what can be construed as meaningful co-creation. People experiencing socioeconomic deprivation, youth, migrants and ethnic minorities are often over-represented among these seldom-heard groups [37, 38]. Their involvement in co-designing the evaluation of co-creation activities is crucial to assess the need for adaptation and jointly devise strategies to implement change [39].

In this paper, we describe a research protocol designed to implement, evaluate and strengthen co-creation methodologies within a project aimed at improving knowledge on mental health promotion and prevention interventions in seven European countries – the ADVANCE project. We will engage stakeholders from diverse social and cultural backgrounds in co-creating the project and adopt an approach to co-creation evaluation that is both participatory and formative [35, 39, 40].

## Methods

### The ADVANCE project

Our protocol was developed within the context of the Horizon Europe-funded ADVANCE project (https://advancementalhealth.ku.dk) which is running from June 2023 to May 2028. The main goal of the ADVANCE project is to demonstrate how mental health promotion and prevention interventions (digital, in-person and combined) from youth to old age can be optimally implemented and scaled up. ADVANCE is a mixed-methods project with an overarching interdisciplinary focus on structural injustice, human rights and stigma prevention. A key element of the project is a co-creation process building on participatory research activities. We adopted the Mental Health Europe (MHE) definition of co-creation as “a collaborative approach involving all actors in mental health working together on an equal basis to develop and implement policies, services and communication that foster positive mental health according to a psychosocial model and human rights-based approach” 41. This process will be implemented throughout the lifetime of the research project.

Broadly, the ADVANCE project consists of: (1) a structured co-creation process involving service end-users, members of mental health umbrella organisations, healthcare practitioners, and decision-makers, (2) an interrelated set of intervention studies with diverse groups in situations conferring vulnerability (from youth to old age), and (3) scaling-up strategy development. Participants in the intervention and scaling studies include people exposed to different sources of adversity in seven European countries, namely: youth with climate change-related distress in Germany; socioeconomically disadvantaged young adults in Lithuania; working adults in highly digitalized work environments in the Netherlands; adult migrants in Italy, Denmark and Finland; and older adults in Switzerland.

The interventions included in the ADVANCE project were strategically pre-selected at the proposal stage on the basis of a set of guiding principles (Table 1). These principles reflected the project’s overarching aim of addressing persistent gaps in the field of mental health promotion and prevention – specifically, the limited emphasis on implementation research, fragmented efforts by condition and population group, and the underuse of scalable, stigma-reducing approaches. The selected interventions target psychological processes and strengths that promote mental health more broadly and help prevent a range of mental health concerns. This decision to avoid interventions tailored to specific mental health conditions stems from the understanding that mental health issues often intersect and cannot be addressed in isolation. Moreover, focusing solely on one condition may perpetuate labelling and stigma, potentially hindering engagement in interventions. Building on so-called third wave cognitive behavioural intervention techniques [42], these interventions emphasize processes such as acceptance, compassion, mindfulness and attention. Developing skills to enhance these processes has shown effectiveness in mitigating a wide range of mental health concerns [43].

**Table 1:** Overview of participating countries, target groups and interventions

Country	Target Groups	Interventions	Available evidence	Aim of intervention study
Germany	Youth affected by climate change-related distress	CliMACT	Kirby, 2017 [44]; Schick, 2021 [45], Reininghaus, 2023 [46]	Assess effectiveness of CliMACT, an adapted version of EMIcompass
Lithuania	Young adults affected by socioeconomic adversity	STARS	Hall, 2022 [47]	Assess efficacy of STARS
the Netherlands	Adults in digitalised work settings	ASCEND and DWM	WHO, 2020 [48]	Assess effectiveness of ASCEND and DWM
Italy	Adults with a migrant background	SH+ + booster session versus DWM + booster session	Tol, 2020 [49]; Acarturk 2022 [50], Purgato, 2021 [51]; WHO, 2020 [48]	Assess optimal implementation formats and booster session for long-term prevention
Denmark	Adults with a migrant background	SH+	Tol, 2020 [49]; Acarturk 2022 [50], Purgato, 2021 [51];	Assess optimal implementation formats
Switzerland	Older adults	SH+ and COG	Tol, 2020 [49]; Acarturk 2022 [50], Purgato, 2021 [51];	Assess effectiveness of combined stress management and cognitive training
Finland (scaling-up country)	Adults with a migrant background	SH+	Tol, 2020 [49]; Acarturk 2022 [50], Purgato, 2021 [51];	N/A

CliMACT, Climate Mind and ACT; STARS, WHO’s Sustainable Technology for Adolescents and Youth to Reduce Stress; ASCEND, WHO’s Advancing Supervisor Capabilities for Mental Health at Work; DWM, WHO’s Doing What Matters in Times of Stress; SH+, WHO’s Self-Help Plus; COG, Cognitive Training

The selected interventions also share several additional features: (i) they have prior evidence of effectiveness, including from trials conducted by ADVANCE partners and/or the World Health Organization; (ii) are suitable for digital, in-person or hybrid delivery formats; (iii) are adaptable for use across age groups and country contexts; and (iv) are built on technological innovations, particularly in mobile health, offering strong potential for cost-effective scale-up. Rather than selecting the interventions through the co-creation process itself, co-creation was used to tailor implementation strategies in each context – including decisions about delivery modes, settings and target subgroups.

### Co-creation process

**Fig. 1:**
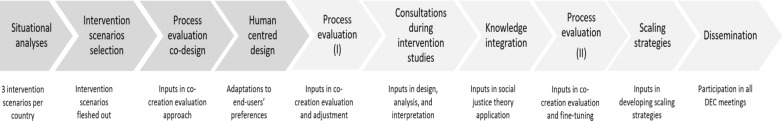
Overview of co-creation phases throughout ADVANCE

ADVANCE’s co-creation activities will run throughout the entirety of the project and will be led in collaboration with Society Advisory Groups (SAGs). In this protocol, we describe the roles of the SAGs who will be involved in a meaningful and systematic way in all key phases of the project (Fig. 1), detail the co-creation activities pertaining to the first year of the project (July 2023 to June 2024), and introduce our co-creation process evaluation approach, which is in its early stages. Other co-creation phases will be described elsewhere (e.g. human-centred design).

The first year in the co-creation process entailed situational analyses and the selection of intervention scenarios through scenario-based workshops. They were designed to involve SAGs, and other stakeholder representatives, in decisions about specific aspects of the mental health prevention and promotion interventions that will be implemented and tested in the intervention countries during the project, namely their target groups, recruitment sites and strategies and intervention delivery modes. SAGs will also participate in the evaluation of the project’s co-creation activities, which is underway and will continue until the end of the project. We will undertake a participatory and formative co-creation evaluation approach, whereby stakeholders will engage in the co-design of the evaluation process and in identifying the adjustments required to meet co-creators’ needs and preferences. The phases of the project that have been already completed are highlighted in darker grey in Fig. 1.

#### Society Advisory Groups

Society Advisory Groups (SAGs) comprise a diverse set of people representing end-users, healthcare practitioners, decision-makers and national mental health umbrella organisations. They aim to ensure that stakeholders’ perspectives are embedded in all aspects of the project through consultation and input on decision-making regarding research activities.

Each SAG is coordinated by two local facilitators: a representative of relevant end-user groups and someone with a scientific background. SAG facilitators were provided with a 1-day face-to-face training by MHE in October 2023 in Brussels. The training is available in a standardised format at MHE’s co-creation toolkit [52], but its staff adapts each training to meet the distinct needs of co-creators involved in different initiatives. In the case of ADVANCE, the training aimed to promote mutual learning and liaison among facilitators and to strengthen their skills to translate co-creation knowledge into practice. It emphasised the principles of solidarity, equity and respect and provided participants with tools to address arising power imbalances and ensure that all views are equally valued. In addition, guidance was provided on how to select and engage SAG members locally, including the importance of defining co-creation roles clearly, addressing SAG members’ needs (e.g. translation and mobility) and managing future expectations (e.g. time investment and compensation). Facilitators were also informed about recruitment strategies and the need to guarantee gender balance and age, ethnic, geographic, and work setting diversity, as well as members’ commitment to a psychosocial approach to mental health and to work within a multi-stakeholder group.

Following the training, seven SAGs were established: one in each of the six countries where interventions and implementation strategies will be evaluated prior to developing scaling strategies (Denmark, Germany, Italy, Lithuania, the Netherlands, and Switzerland) and one in the country where the focus is immediately on scaling (Finland). Each SAG includes 10–12 representatives. End-users and members of umbrella organisations were recruited by local teams and through MHE affiliate organisations in each country. Practitioners (i.e. mental health and social services providers), decision-makers and regulators were purposefully selected by local teams and through the WHO and Mental Health Europe (MHE) networks. When members withdraw from SAGs, local teams, in collaboration with the remaining SAG members, undertake efforts to recruit new participants whose backgrounds and experiences closely reflect those of the departing stakeholders.

#### Year 1 co-creation activities

**Fig. 2:**
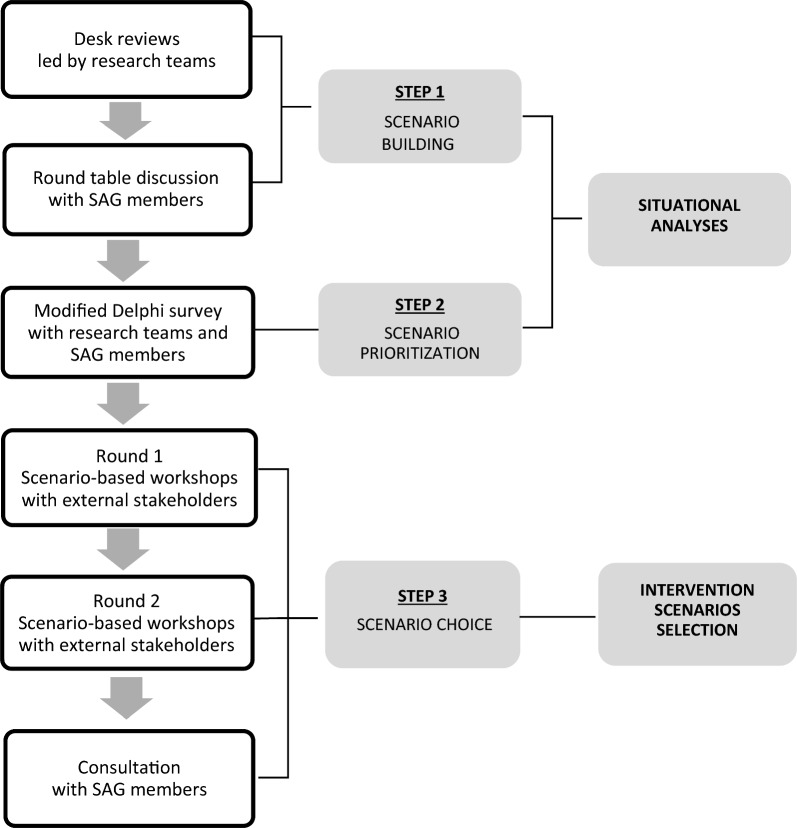
Co-creation steps guiding the situational analysis and selection of intervention scenarios

The aim of co-creation activities in year 1 of the ADVANCE project was to develop an intervention scenario for the six intervention studies and the seventh study site (where scaling-up strategy development will be based on previous, existing findings) starting in July 2023 (Table 1). Using a mixed-methods approach, co-creation activities in year 1 encompassed collaboration between local research teams and the SAG members to: conduct a desk review; identify potential intervention scenarios; and prioritise three intervention scenarios using a modified Delphi panel in each of the intervention study countries (i.e. situational analysis). The three scenarios receiving the highest priority were presented for discussion at scenario-based workshops held with representatives of stakeholders in the field of mental health promotion and prevention (i.e. end-users, patient organisations, health care professionals and decision-makers) external to the project, hereafter referred to as external stakeholders. Following a discussion on the feasibility and relevance of each intervention scenario, participants sought to reach a consensus on the preferred scenario for implementing mental health interventions. They were also asked to propose strategies to mitigate stigma during recruitment and implementation of the interventions. An overview of the different activities conducted in year 1, which are part of the interrelated phases “situational analysis” and “intervention scenarios selection”, is provided in Fig. 2. Below, we discuss the activities according to the three steps displayed in the middle of Fig. 2.

### Step 1. Scenario building

With the main objective of documenting current challenges and informing the scientific community and public authorities about new and emerging risks to mental health in a changing Europe, seven desk reviews were carried out by local research teams followed by a round-table discussion with SAGs to incorporate their expert views and supplement missing information (see Mack et al., 2025) [53]. Desk reviews followed the scoping review protocols proposed by Levac et al. [54] and by Arksey and O’Malley [55]. They were also informed by the toolkit on mental health needs assessment in humanitarian settings developed by the WHO and the United Nations High Commissioner for Refugees UNHCR (2012) and by related key desk review parameters discussed by Greene et al. [56].

In the six intervention countries, rapid desk reviews synthesized academic and grey literature documenting how climate change, digitalisation, migration, socioeconomic stressors and ageing in Europe are affecting the mental health of pre-selected target populations in these countries. Evidence on the groups most vulnerable to stigmatisation within these populations and strategies for stigma prevention or reduction were also gathered from literature whenever available.

The findings of the desk reviews were synthesised and streamlined with the ultimate aim of enabling the design of a set of potential intervention scenarios (at least five scenarios per country). Each intervention scenario was designed around three main aspects: (1) target groups (i.e. subgroups within the pre-selected target population who are most vulnerable to the mental health threat under investigation in each country); (2) recruitment strategies (i.e. choice of location and process for recruitment); and (3) intervention delivery modes (i.e. online, in-person or hybrid).

Preliminary results of the desk reviews were shared with the SAGs through presentations in meetings, where input was gathered and integrated into the desk review as part of the consultation stage. On the basis of their experiential knowledge, SAG members helped to clarify or added information on aspects not covered by the literature reviewed. This step also provided an opportunity for SAG members to add valuable information to refine the intervention scenarios identified or to suggest new scenarios to the list of scenarios initially proposed by the research teams.

In the scaling-up country, Finland, the desk review focused on identifying strategies to enhance the societal impact of an intervention proven effective in prior research: SH+ for migrants [51]. It consolidated academic and grey literature reporting on current mental health support services for migrants in Finland, as well as municipalities’ needs and available resources. This information was integrated to deliver evidence of direct relevance to the future planned development of scaling strategies on the basis of Osterwalder and Pigneur’s Business Model Canvas strategic management approach [57]. The initial draft of the business plan resulting from the desk review was shared with the SAG during the consultation stage, allowing them to contribute valuable insights to refine the key components of the plan.

### Step 2. Scenario prioritization

Modified Delphi surveys were undertaken in the six intervention countries to enable the selection of three intervention scenarios to be presented in the next step – the scenario-based workshops. While the desk reviews aimed to surface all relevant potential intervention scenarios, the Delphi surveys enabled SAGs and local research team members to engage in the prioritisation of the three most relevant intervention scenarios for discussion in the scenario-based workshops.

The Delphi survey method embodies the principles of inclusivity and diversity inherent in co-creation endeavours. The Delphi survey method is a group facilitation technique that uses a structured and iterative approach to gather insights and gain consensus from a diverse group of experts on a particular topic or problem [58]. Delphi methodology has been applied across various disciplines and is often used in strategic decision-making in public health. It is characterized by an iterative nature, anonymity, controlled feedback and statistical aggregation of panel responses [59, 60]. This approach is particularly valuable for mitigating group conformity (known as groupthink) and avoiding the so-called halo effect, where undue weight is given to the opinions of dominant or higher-ranking individuals within the group [59, 61].

A two-round modified version of the Delphi survey method was conducted in each intervention country, utilizing web-based platforms compliant with the General Data Protection Regulation (GDPR). Panel participants were purposefully sampled to include all members of the local research teams and of the SAGs in each intervention country. This approach aimed to include participants with a diversity of backgrounds, perspectives and affiliations, facilitating the capture of a wide range of opinions and insights, and ensuring the validity and reliability of the process. In contrast with a conventional Delphi study, the initial stage of this study did not include an open-ended round to gather qualitative data. Instead, this modified Delphi survey departed from the predefined set of potential intervention scenarios designed upon the results of the desk reviews.

Round 1: To prioritize scenarios, participants were asked to rank each intervention scenario in order of importance using a 6-point Likert scale. They were prompted with the question: “Please rate the intervention scenarios below according to the importance of their implementation”. The Likert scale ranged from 1 (not important at all) to 6 (extremely important). This scale omitted a midpoint to discourage neutral responses, which could potentially affect the reliability and validity of the results if the midpoint is used as a “dumping ground” [60, 62]. Participants were also given the opportunity to provide any additional thoughts, suggestions or questions they may have through an open-ended question at the end of the questionnaire.

Round 2: Participation in this round was extended exclusively to those individuals who had completed round 1. Invitations for round 2 included feedback on each participant’s individual ratings from the previous round, along with summary statistics representing the collective ratings of all participants and (anonymised) responses to the open-ended question. Participants were prompted to reflect on this information and revaluate each intervention scenario. The same question and rating scale utilized in round 1 were used. Participants had 8 days to complete each questionnaire, and two reminder emails were sent before the deadline.

Descriptive statistics, including median scores and interquartile ranges (IQR), were computed to assess the relative importance of each item on the basis of the entire response sample. All panellists’ answers were weighed equally. Although no consensus exists concerning evaluation criteria, most studies use levels of agreement between 60% and 80% [63]. Therefore, adopting a conservative approach, consensus for intervention scenarios prioritization was defined a priori as ≥ 75% of the participants’ ratings for a determined scenario option falling within the two higher categories (5, very important; 6, extremely important) on the 6-point Likert scale.

### Step 3. Scenario choice

The selection of a scenario for implementation in each intervention country was performed using scenario-based workshops during which participants were also asked to propose strategies to reduce stigmatisation during the implementation of mental health promotion and prevention interventions.

The scenario-based workshops method is a group facilitation technique used to elicit expert opinions and foster consensus among a diverse group of stakeholders about a set of scenarios or alternative representations of the future. The scenarios provide an outline of possible futures without claiming to be complete or fully accurate [64]. By identifying and incorporating trends and uncertainties, the scenarios help to develop forecasting activities and to reduce propensity for error in decision-making due to overconfidence or tunnel vision [65]. Scenario methodologies drawing on participatory workshops can offer an innovative approach to the implementation process of mental health interventions. Originally circumscribed to forecasting studies, scenario methodologies have proven effective in promoting dialogue, consensus-building and facilitating strategic planning across diverse areas [65, 66]. They have been increasingly used in healthcare contexts, including for mental health [64]. However, their application has been mainly limited to the prospective planning of health services in dementia care and exploring possible future scenarios of mental health care from a social perspective [64, 67].

There are several advantages to integrating scenario-based workshops into the co-creation of optimal implementation strategies. First, it allows stakeholders to analyse potential future scenarios, anticipate challenges and identify opportunities, thereby enhancing the adaptability and relevance of intervention strategies [64]. Second, by considering multiple scenarios, it cultivates a more nuanced understanding of the complex dynamics within the mental health domain, enabling stakeholders to make informed choices that account for various determinants [68]. These workshops can also foster a sense of co-ownership and accountability among stakeholders, as they collectively explore innovative solutions to approach complex mental health challenges. Moreover, it encourages proactive thinking in a safe environment where sensitive or emotionally charged issues, beliefs and perceptions can be brought forward without fear of judgement, ridicule or disregard [69].

Scenario-based workshops typically start with the presentation of the scenarios to stakeholders followed by discussion about their feasibility, relevance, advantages and disadvantages within a first round of independent in-group workshops. Subsequently, the facilitator analyses and compiles the stakeholders’ feedback about each scenario and shares it with participants in the final mixed-group round of workshops. In that last round, participants are asked to select a scenario for intervention [70].

This step in our study entailed a two-round scenario-based workshop method that was used to bring about stakeholders’ expertise in devising strategies to minimise stigmatisation and build consensus over the most relevant intervention scenario. Participants included representatives of four groups of stakeholders external to the project, namely end-users, members of umbrella organisations, healthcare practitioners and decision-makers (e.g. politicians and regulators). These representatives were purposefully sampled and recruited by local research teams in collaboration with gatekeepers and through WHO’s and MHE’s networks and affiliate organizations. Invitations for participation were sent via email, including an Information Sheet and an Informed Consent Form. Participants signed the consent form via email or in person before the scenario-based workshops. Each intervention country recruited between 10 and 17 participants, with a minimum of 1 and a maximum of 6 participants per stakeholder group.

The scenario-based workshops were held in person at accessible locations for participants, moderated by a research team member with the support of an observer (also a research team member), who was in charge of taking notes (e.g. nonverbal behaviour, key conclusions) during the workshops. They were conducted in the local language or in English, depending on what was viewed locally as most appropriate.

The scenario-based workshops consisted of two rounds: (a) the first round involved two workshops, one for end-users and mental health umbrella organization representatives (6–10 participants) and another for mental healthcare practitioners and decision-makers (6–10 participants); and (b) the second round featured a mixed-group workshop with randomly selected representatives from all four stakeholder groups (end-users, umbrella organization members, healthcare practitioners and decision-makers), totalling 5–10 participants. By holding two separate workshops in the first round, this approach aimed to avoid power imbalances and encourage sharing diverse perspectives. In the first round, participants discussed the advantages and disadvantages of each intervention scenario and devised strategies to minimise stigmatisation during the implementation of the intervention. In the second round, participants reviewed and discussed the identified advantages and disadvantages, selected an intervention scenario and finalized the list of stigma reduction strategies. The workshops were recorded, transcribed and translated from the local language into English when necessary. The results of the scenario-based workshops were discussed by the research teams with SAG members who, in some instances, made suggestions to refine specific aspects of the scenarios (e.g. related to recruitment sites).

#### Co-creation evaluation

The evaluation of ADVANCE’s co-creation process will follow a participatory and formative approach. Its main aim is to promote the quality, relevance and acceptability of co-creation activities by monitoring ongoing practices, identifying issues for improvement and co-developing strategies to address them. To this end, local research teams and SAG members were involved in co-designing the evaluation process together with the coordinators of the project’s co-creation work package (WP). They agreed to conduct a longitudinal qualitative study drawing on semistructured interviews with co-creators at two points in time. This design was selected to capture shifts in co-creators’ experiences and perspectives over time and to facilitate adaptation moving forward. The interview guide will be co-developed with co-creation representatives through a World Café.

**Fig. 3:**
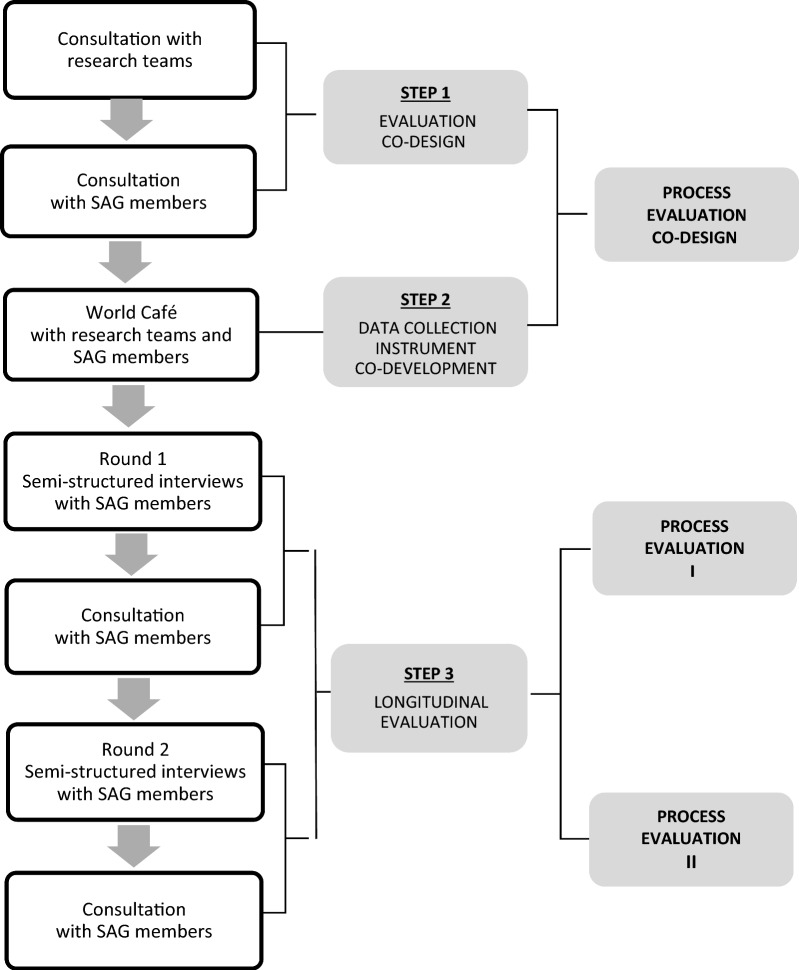
Steps guiding the co-design, co-development and implementation of co-creation evaluation

The World Café will be conducted during year 2 of the project, after which the first wave of interviews will follow. Adaptations deriving from first-wave evaluation activities will be examined in the second wave of interviews in year 4. The results will be used to fine-tune co-creation practices leading to project completion. Figure 3 shows an overview of the evaluation activities planned within the phases of “process evaluation co-design”, “process evaluation I” and “process evaluation II”. Next, we provide further detail on the three specific steps guiding the co-creation process evaluation.

### Step 1. Evaluation co-design

All partner countries were involved in co-designing the evaluation of co-creation activities through a set of online bimonthly meetings and email exchanges with the project’s co-creation WP coordinators between September and October 2024. A proposal detailing different elements of a potential research design (e.g. methods, participant groups and core components of co-creation to be evaluated) was shared in the first meeting. Queries and feedback were gathered from the research team members present. Other team members shared input through email communication after receiving the meetings’ memos.

To perform the evaluation, a longitudinal qualitative research design based on interviews with co-creators was suggested and accepted. Participants agreed to focus the evaluation on three co-creation components, namely: strengths of and challenges to the co-creation process; co-creators’ concerns and motivations to engage; and co-creation impact on interventions and co-creators. To ensure sensitivity and impartiality, it was proposed that the interview guide be co-developed by co-creators’ representatives on the basis of one of the following methods: World Cafés, focus groups or written feedback. This last proposal generated the most discussion, concerning feasibility. Although the majority favoured a World Café, it was argued that having research teams run World Cafés locally would require too many resources and time. A team member’s proposal to conduct one single World Café, combining participants from all partner countries and led by the WP coordinators, finally gathered consensus towards implementation.

In the first round of interviews, participants will be asked if they would like for other methods to be used in the evaluation of co-creation activities as a supplement to the longitudinal interviews. Employing surveys, for example, may allow for more periodic, less burdensome data collection and monitoring.

### Step 2. Data collection instrument co-development

The World Café is a participatory method that entails a conversational process set to enable constructive dialogue and the gathering of individuals’ ideas into a comprehensive message or output. It strives to provide a welcoming environment (i.e. a café-style atmosphere) where participants can speak freely and without the imposition of having to reach a consensus [71]. In an in-person World Café, participants can hold conversations in a general assembly format or in small groups gathered around separate tables. Participants switch between tables over several rounds, with the exception of a table host who welcomes the new group of participants to the table. In each round, notes of the conversations are made using paper tablecloths. This fosters the cumulative sharing of ideas and the coupling of different perspectives over specific topics. At the end of the session, a general assembly is held to share the views gathered with the whole group and prioritise the most pressing issues [72].

In our study, the World Café will involve co-creators based in seven countries. For this reason, it will be hosted online. Participants will include one SAG member and one team member per partner country who will be invited to co-develop the interview guide. All SAG members who agree to participate in the World Café in English will be eligible for participation and sampled through random selection. Research teams will assign a participant of their choosing. In total, 14 participants will be recruited to take part in the World Café after providing signed consent by email. Participants’ permission to audio-record the interview will be requested.

The World Café will be hosted by the co-creation WP coordinators supported by two observers. The Voice over Internet Protocol (VOIP) platform Zoom will be used to run the session online. Zoom breakout rooms will be used as substitutes for the café tables to enable a quiet setting for small group discussions. Observers will be present in the breakout rooms. They will take notes and help summarise the main ideas for the general assembly but will not participate in the discussions.

To emulate the friendly atmosphere of an in-person World Café, participants will be encouraged to take a beverage and snacks with them. A visual background image alluding to a café will be created and displayed during the online session using Conceptboard, a digital pinboard containing virtual Post-its that can mimic the role of tablecloths by allowing participants to write down their ideas [72].

The World Café will initiate with the presentation of the ground rules for participation and purposes of the session. A general assembly will follow where the hosts will introduce the abovementioned co-creation evaluation core components (i.e. co-creation process strengths and challenges, co-creators’ experiences and perspectives, and co-creation impact). Participants’ feedback on whether those components can be used as signposts to co-develop the interview guide, or if they have alternative proposals, will be asked. They will also be briefly prompted on how to design interview questions. Subsequently, participants will be invited to enter breakout rooms for small group discussions. The rounds of small group discussions will match the number of interview guide components (e.g. three rounds will be held if no changes are proposed).

Participants will be randomly assigned to the breakout rooms by the hosts, after which they will be invited to select a table host. Each small group discussion round will last 15 min. Participants will be asked to contribute questions on each interview guide component, building on the previous group’s contributions. The questions proposed will be jotted down using the GDPR-approved software Conceptboard [73]. A screenshot of the digital pinboards filled in each breakout room will be made. The World Café hosts, participants and observers will reconvene at a final assembly to share the questions gathered and eliminate redundancies.

The screenshots of participants’ contributions made during the small group discussions and the assembly will be reviewed and streamlined to refine the interview guide. The observers’ notes will be subject to thematic analysis to identify key topics and patterns in participants’ experiences concerning the guide co-development and the overall co-creation process, which may be helpful when framing prompts to assist in interviewing. A summary of the results of the World Café will be sent to all participants.

### Step 3. Longitudinal evaluation

Longitudinal in-depth semistructured interviews employing the co-created interview guide will be carried out online at two time points to evaluate the co-creation process. The first wave of interviews will be conducted in year 2 of the project, focusing on the benefits of and challenges to co-creation implementation, co-creators’ motivations and concerns, co-creation impact and actions needed to improve co-creation relevance and acceptability. Interview results will be subsequently shared with SAG members and research teams to help co-devise strategies to implement adjustments to the co-creation process.

The second wave of interviews will take part in year 4, delving into the previous topics as well as into participants’ perspectives about the adaptations undertaken following the first set of interviews. Consultation with SAG members and research teams will unfold once again to support fine-tuning of ongoing co-creation activities and facilitate reflection on the lessons learned.

Both interview waves will be conducted using the Zoom platform. Participants will be purposively sampled to include the two SAG facilitators of each partner country. To ensure diversity of views, another two SAG members will be invited to participate. All SAG members who agree to participate in an interview in English will be listed as eligible and randomly selected for participation. Invitations will be sent by email together with an information sheet and an informed consent form, specifying their desired participation in both interview waves. Four SAG members per partner country are expected to take part in the interviews entailing a total of 28 participants. Participants will be interviewed by the co-creation WP coordinators after returning the signed consent form. Permission to audio-record the interview will be requested.

The interview audio recordings will be transcribed verbatim. The transcripts will be analysed using thematic analysis [74]. A deductive and inductive approach will be employed to identify key themes and patterns in participants’ views about, experiences with and attitudes towards the process of research co-creation with support of the NVivo 14 software.

## Discussion

The co-creation process outlined in this protocol aims to address the pressing need for strengthening availability of evidence-based mental health promotion and prevention strategies in Europe. The ADVANCE project aims to contribute both new evidence (i.e. new effectiveness evaluation studies for age groups, vulnerabilities and intervention approaches where evidence of effectiveness is lacking) as well as conduct implementation research for interventions for which evidence on effectiveness is already available. Through research carried out in co-creation with relevant stakeholders, and a multi-country scope, the project hopes to offer results from a diverse and comprehensive perspective, allowing for an improved understanding of which implementation concerns for mental health promotion and prevention interventions are more generalizable, and which concerns are more contextually determined.

Our co-creation process aims to strengthen implementation and research on mental health promotion and prevention interventions in several ways. First, we would like to uphold the fundamental principle of co-creation of bringing together individuals with different skills and expertise to work together in an equal basis, ultimately generating new knowledge, and shaping policies and interventions with positive societal impact [22]. Second, it aligns with the broader paradigm shift towards complexity-informed health promotion. A recent review highlights the need to integrate health promotion and co-creation methodologies to effectively address the complex and dynamic nature of health issues [75]. By adopting a co-creation framework, we acknowledge the diverse factors influencing mental well-being, including socioeconomic, cultural and environmental determinants, enabling us to develop contextually relevant and adaptable interventions responsive to the needs of diverse populations across an ever-changing Europe. Third, co-creation has been known to foster adaptability, a critical factor for the success and sustainability of social change initiatives, including mental health promotion interventions [76]. The ability of interventions to adapt to the needs and contexts of target populations and settings is essential for their adoption and long-term impact. By embracing co-creation as a dynamic and iterative process, stakeholders can harness the adaptive capacity of interventions to address evolving mental health challenges and societal contexts. Fourth, our co-creation approach will extend beyond the initial stages of the project to include later phases such as intervention evaluation, dissemination and scaling strategy development. This comprehensive involvement may help to fill an evidence gap identified in a previous systematic review that found that only 11% of the studies reviewed (*n* = 26) applied co-creation in the development of the evaluation process [77]. Finally, we will engage co-creators in co-designing the evaluation of co-creation activities and in adapting co-creation strategies as the project unfolds. Here, there is an even more substantive limitation in the evidence available, with less than 3% of the studies reviewed (*n* = 54) by Longworth and colleagues [27] undertaking participatory evaluation of co-creation research. Our study thus responds to calls for knowledge generation that can substantiate the development and implementation of participatory and formative co-creation evaluation approaches [27, 31, 78].

While we expect benefits of the co-creation process, both for stakeholders and for research outcomes [79, 80], we also anticipate several hurdles to navigate. These include reconciling diverse values and priorities; addressing power dynamics and conflicts in stakeholder motivations; managing expectations; accommodating changes in established research methodologies and timeframes; preventing researchers’ tokenistic attitudes; and devoting considerable time to nurturing relationships [75, 80, 81]. A specific tension that we foresee involves predefined parameters of the research proposal, including broad target groups, mental health risks and associated interventions. These are broad parameters over which the SAGs and others involved in the co-creation process have little influence, thus creating an unavoidable power dynamic. As discussed above, the co-creation activities in year 1 are aimed at specifying the details for intervention scenarios. This is a tension that is challenging to overcome in the context of research proposals funded through calls with pre-set conditions for research content. Nevertheless, continuous efforts are being made to prioritize transparent communication and ensure a common understanding of stakeholders’ roles from the onset and all along the process, including the limitations deriving from previously defined aspects (e.g. SAG facilitators were made aware of these limitations during the training held at the project start, which will continue to be a topic of discussion throughout implementation).

We also foresee several challenges in conducting this study, particularly owing to its multi-site and participatory nature. Effective communication will be paramount to ensure seamless coordination with the local teams. To address this, we implemented a bi-weekly teleconference schedule to facilitate regular updates on the study’s progress across all sites. We also seek to juxtapose pressing demands for local research teams’ time and resources associated with the co-creation process with continuous support and guidance from the co-creation WP coordination team, which employs a hands-on approach to developing research materials (e.g. protocols) and facilitating implementation (e.g. data collection through World Café) in dialogue and collaboration with local teams. A further significant challenge concerns the methodological demands inherent in comparative co-creation research. Co-creation is a systemic, nonlinear, relational process that frequently involves context-specific adaptations. While standardised research protocols are established, methodological adjustments across research sites have been often necessary to accommodate participants’ needs and preferences. These adaptations can create tensions between the scientific rigour required for comparative analysis and the flexibility essential for meaningful co-creation. However, such adaptability is key for fostering engagement and ensuring the sustainability of research. Rather than viewing adaptation solely as a limitation undermining comparison, we seek to collectively reflect on and detail its potential shortcomings while recognising it as an intrinsic perk of participatory research.

Furthermore, our innovative methodology combining qualitative and participatory methods reflects a commitment towards minimising inequities and preventing participation fatigue [78]. As Agnello and colleagues [82] point out, participatory methods such as World Cafés and scenario-based workshops have yet to gain traction in academic co-creation research, but they carry substantial potential to help build trust, address power imbalances and reduce disengagement. Finally, we will continue to strive to keep the SAGs involved and motivated throughout the duration of the project by eliciting their views, identifying drawbacks and areas for improvement, and adapting co-creation activities to ensure meaningful collaboration and a sense of shared ownership. The decision to undertake participatory and formative co-creation evaluation is a key step in this direction. Additional measures to foster co-ownership within our project include early dialogue about and clarification of roles and responsibilities among SAGs and local research teams and creating opportunities for critical reflection and transparent discussion of expectations and misunderstandings. We also emphasize joint decision-making regarding the dissemination of outputs and co-authorship of academic and non-academic contributions (e.g. policy and community briefs). Importantly, our conception of co-ownership extends beyond outputs to include capacity building and long-term benefit-sharing, which we will seek to assess and promote as part of the project evaluation activities.

## Conclusions

The co-creation process described in this protocol represents a potential advancement in mental health promotion research and practice, where co-creation has been limited especially in intervention evaluation stages. By systematically involving stakeholders in problem analysis and the design and selection of the most suitable intervention scenario, this protocol hopes to strengthen the possibility that interventions are not only evidence-based but also culturally sensitive, acceptable, ethically aligned and seamlessly integrated into existing practices. Sustaining the involvement of stakeholders throughout the project lifecycle is crucial to reach these goals. To this end, this protocol additionally includes a participatory evaluation component. This evaluation seeks to generate critical insights into how co-creation is experienced by different stakeholders and how it shapes implementation processes and outcomes. By studying the co-creation process itself, we aim to make a methodological contribution to participatory research, while also identifying ways to make implementation more responsive, inclusive and contextually grounded.

Our co-creation approach, rooted in the principles of equity, and ongoing participatory evaluation and adaptation, envisions the redistribution of power and acknowledges the invaluable insights of individuals with lived experience in driving transformative change in mental health research and practice. Ultimately, we hope results will yield practical insights and high-level recommendations to advance co-creation of mental health research, inform policy decisions, reduce stigma and enhance mental well-being across Europe and beyond.

## Data Availability

No datasets were generated or analysed during the current study.

## Abbreviations

SAG: Society Advisory Group
WP: Work package
VOIP: Voice over Internet Protocol
